# Cell type-specific effects of p27^KIP1^ loss on retinal development

**DOI:** 10.1186/s13064-017-0094-1

**Published:** 2017-09-20

**Authors:** Mariko Ogawa, Fuminori Saitoh, Norihiro Sudou, Fumi Sato, Hiroki Fujieda

**Affiliations:** 1 Department of Anatomy, School of Medicine, Tokyo Women’s Medical University, 8-1 Kawada-cho, Shinjuku-ku, Tokyo, 162-8666 Japan; 2 Department of Anatomy, School of Medicine, Toho University, 5-21-16 Omorinishi, Ota-ku, Tokyo, 143-8540 Japan

**Keywords:** Cyclin-dependent kinase inhibitor, p27^KIP1^, Retinal development, Ectopic cell cycle reentry, Cone death

## Abstract

**Background:**

Cyclin-dependent kinase (CDK) inhibitors play an important role in regulating cell cycle progression, cell cycle exit and cell differentiation. p27^KIP1^ (p27), one of the major CDK inhibitors in the retina, has been shown to control the timing of cell cycle exit of retinal progenitors. However, the precise role of this protein in retinal development remains largely unexplored. We thus analyzed p27-deficient mice to characterize the effects of p27 loss on proliferation, differentiation, and survival of retinal cells.

**Methods:**

Expression of p27 in the developing and mature mouse retina was analyzed by immunohistochemistry using antibodies against p27 and cell type-specific markers. Cell proliferation and differentiation were examined in the wild-type and p27-deficient retinas by immunohistochemistry using various cell cycle and differentiation markers.

**Results:**

All postmitotic retinal cell types expressed p27 in the mouse retinas. p27 loss caused extension of the period of proliferation in the developing retinas. This extra proliferation was mainly due to ectopic cell cycle reentry of differentiating cells including bipolar cells, Müller glial cells and cones, rather than persistent division of progenitors as previously suggested. Aberrant cell cycle activity of cones was followed by cone death resulting in a significant reduction in cone number in the mature p27-deficient retinas.

**Conclusions:**

Although expressed in all retinal cell types, p27 is required to maintain the quiescence of specific cell types including bipolar cells, Müller glia, and cones while it is dispensable for preventing cell cycle reentry in other cell types.

**Electronic supplementary material:**

The online version of this article (10.1186/s13064-017-0094-1) contains supplementary material, which is available to authorized users.

## Background

During development of the CNS, precise coordination of progenitor cell proliferation and cell cycle exit is essential for generation of appropriate number of neurons. Cell proliferation is driven by cell cycle-specific cyclins assembled with their catalytic partners, cyclin-dependent kinases (CDKs). The activity of cyclin/CDK complexes is regulated by CDK inhibitors, which inhibit cell cycle progression and promote cell cycle exit [1, 2]. Recent evidence has further revealed that CDK inhibitors promote differentiation independently of their ability to regulate CDK activity [3–5]. However, much of the knowledge concerning the role of CDK inhibitors comes from the results of in vitro studies using non-neuronal cells, and whether CDK inhibitors have equivalent roles in vivo in the context of CNS development or whether they function in a cell type-specific manner remain largely unexplored.

The retina is an ideal model of the CNS to gain insight into these questions as it has a relatively simple structure consisting of only seven major cell types, which can be clearly identified by position and specific markers. Multipotent retinal progenitors divide extensively during development and lose proliferative capacity as they withdraw from the cell cycle and differentiate into specific cell types. Yet, how retinal cells exit the cell cycle and maintain the quiescent non-proliferative state is only partially understood. Several types of CDK inhibitors have been detected in the developing retina, of which p27^KIP1^ (p27) is most abundantly and ubiquitously expressed [6]. In the *Xenopus* retina, p27 not only inhibits the cell cycle but also promotes the cell fate of Müller glia [7]. In the rodent retinas, p27 does not seem to have a role in cell type specification, but it promotes the cell cycle exit of retinal progenitors [8, 9]. p27 loss was shown to extend the period of progenitor proliferation [8, 9] and rescue the hypoplastic defects of cyclin D1-deficient retinas [10]. These studies indicated that p27 plays an essential role in controlling the timing of cell cycle exit of retinal progenitors. Recent studies have also revealed that deletion of Rb and its family members in the retina induces ectopic proliferation of differentiating cells, suggesting that the major function of the Rb family in retinal development is to prevent cell cycle reentry of differentiating cells [11–13]. Considering that the Rb family functions downstream of p27, we hypothesized that p27 loss may have effects on differentiating cells, in addition to the previously reported effects on progenitors. To address this issue and delineate more precisely the role of p27 in retinal development, we revisited p27-deficient mice to characterize the effects of p27 loss on proliferation, differentiation, and survival of retinal cells. In contrast with the previous observations, our data suggest that extra proliferation observed in the p27-deficient retinas is mainly due to ectopic cell cycle reentry of differentiating bipolar cells, Müller glia and cones, rather than persistent division of progenitors. Aberrant cell cycle activity of cones was followed by cone death resulting in a significant reduction in cone number in the mature p27-deficient retinas. Our data propose a previously unrecognized cell-specific role for p27 in the maintenance of quiescent state in postmitotic retinal cells.

## Methods

### Animals and tissue preparation

p27^+/−^ mice [14] were obtained from the Jackson Laboratory (Bar Harbor, USA), bred and genotyped by PCR as recommended by the Jackson Laboratory. Animals were maintained under a 12:12 h light/dark photoperiod and sacrificed by decapitation or cervical dislocation in the middle of the light phase at various developmental stages. For immunohistochemistry, the eyecups with the cornea and lens removed were fixed by immersion in 4% paraformaldehyde in 0.1 M phosphate buffer (pH 7.4) for 1 h, rinsed in 15% and 30% sucrose in phosphate buffer, and frozen with dry ice–isopentane. Cryostat sections were cut at 10 μm through the optic disc along the dorsoventral axis and collected on MAS-coated glass slides (Matsunami glass, Osaka, Japan). For RT-PCR, the retinas were dissected and kept frozen at −80 °C until use. All experimental procedures were conducted in accordance with the research protocols approved by the institutional animal care committee of Tokyo Women’s Medical University.

### BrdU incorporation assay

To label mitotic cells in the S-phase, animals received a single injection of BrdU (Sigma, St. Louis, USA, 100 mg/kg body weight, i.p.) 2 h before sacrifice. For birthdating studies, animals were injected twice per day with BrdU and allowed to survive at least 9 days before sacrifice.

### Immunohistochemistry

Immunohistochemistry was conducted as described previously [15, 16]. For BrdU labeling, cryostat sections of the retina were treated with 2 M HCl at 37 °C for 30 min prior to incubation with primary antibodies. Primary antibodies are listed in Additional file 1: Table S1. Secondary antibodies include donkey anti-mouse IgG (Alexa Fluor 488), donkey anti-rabbit IgG (Alexa Fluor 488, 555, 594 and 647), donkey anti-sheep IgG (Alexa Fluor 555), and donkey anti-goat IgG (Alexa Fluor 568), all of which were purchased from Invitrogen (Eugene, USA). Fluorescein-conjugated peanut agglutinin (PNA) (Vector Laboratories, Burlingame, USA) was used to label cones. Fluorescence signals were examined by confocal laser scanning microscope (LSM510 META and LSM710; Carl Zeiss, Germany).

### Cell counting

Cells immunoreactive for specific cell markers were quantitated on vertically sliced retinal sections containing the optic nerve head. Confocal images (at least 10 fields per animal) were obtained from the central retina, defined as 700 μm from the border of the optic nerve head, using a 40× or 63× objective lens (3 animals per stage and genotype). Immunoreactive cells were counted and the density calculated per mm retina. Due to their paucity, pH3-positive cells were counted per whole retinal section. The numbers of cones and horizontal cells were quantitated using whole mount retinas immunolabeled for cone arrestin and calbindin, respectively. Confocal z-stack images, one from each quadrant (142 μm × 142 μm), were captured and cell density calculated per mm^2^ retina (3 animals per genotype). Statistical analysis was conducted by Student’s t-test (*P* < 0.05).

### Quantitative (real-time) RT-PCR

Quantitative RT-PCR was performed using Fast SYBR green Master mix (Applied Biosystems, Foster City, CA) on a 7500 Fast real-time PCR system (Applied Biosystems) as previously described [16]. The list of primers is shown in Additional file 1: Table S2. Data were normalized to *Gapdh* expression and statistical significance analyzed by Student’s t-test (*P* < 0.05).

## Results

### Expression of p27 in the developing and mature mouse retinas

**Fig. 1:**
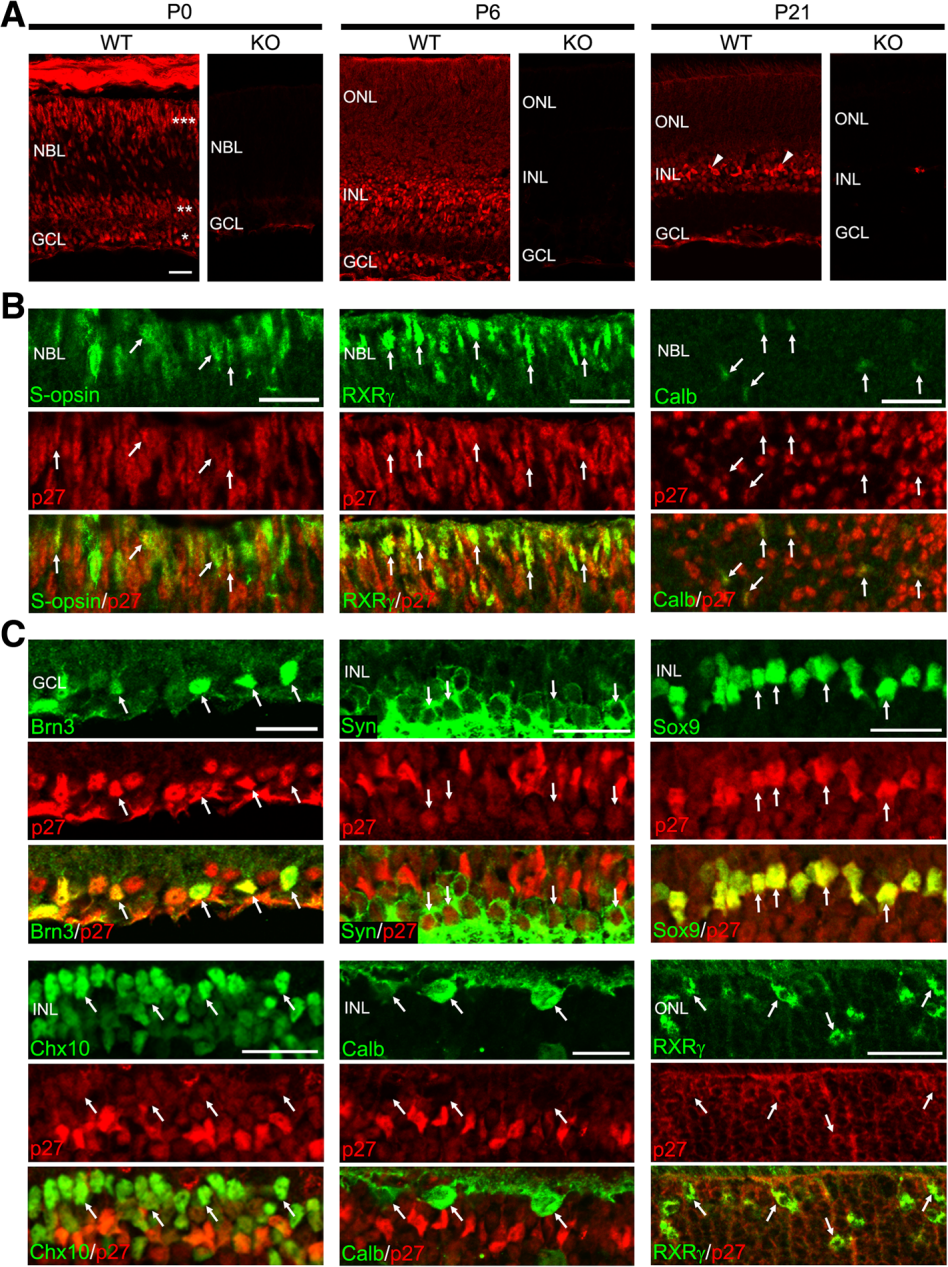
Cellular localization of p27 in the mouse retina during postnatal development. **a** Immunofluorescence for p27 in the wild-type (WT) and p27 knockout (KO) retinas at P0, P6 and P21. At P0, the ganglion cell layer (*), amacrine cell layer (**), and photoreceptor layer (***) are intensely immunoreactive. p27 is detected in all nuclear layers at P6 and P21. Arrowheads indicate Müller glia with intense immunoreactivity. No staining is observed in the KO retinas showing antibody specificity. **b** p27 expression in cones and horizontal cells in the P0 retinas. Double immunofluorescence for p27 in combination with the cone markers S-opsin and RXRγ and horizontal cell marker calbindin (Calb) showing colocalization (arrows). **c** P21 mouse retinas showing expression of p27 in Brn3+ ganglion cells, syntaxin (Syn) + amacrine cells, Sox9+ Müller glia, Chx10+ bipolar cells, calbindin (Calb) + horizontal cells, and RXRγ + cones. Arrows indicate colocalization. NBL, neuroblastic layer; GCL, ganglion cell layer; ONL, outer nuclear layer; INL, inner nuclear layer. Scale bars = 20 μm

We first examined the overall expression patterns of p27 in the developing and mature mouse retinas by immunohistochemistry. At postnatal day 0 (P0), p27 immunoreactivity was observed in the ganglion cell layer (GCL), the inner part of the neuroblastic layer (NBL) containing amacrine cells, and the outer portion of the NBL containing differentiating photoreceptors (Fig. 1a). p27 was detected in most, if not all, cells in the central retina at P6 (Fig. 1a). In the mature retina (P21), p27 immunoreactivity was still present in all nuclear layers of the retina although p27 levels decreased in most retinal cells except Müller glia, which maintained intense p27 labeling (Fig. 1a). Immunolabeling was absent in the p27 knockout (KO) retina, which proved antibody specificity (Fig. 1a).

We then conducted double immunofluorescence for p27 and cell type-specific markers to define the cell types expressing p27. In the mouse retina, ganglion cells, amacrine cells, horizontal cells, cones, and some rods are generated by birth [17]. Although previous reports suggested that p27 is expressed in ganglion cells, amacrine cells and rods in the newborn retina [8, 9], p27 expression in cones and horizontal cells remains unexplored. We thus performed immunolabeling for p27 in combination with the cone markers S-opsin and RXRγ [18] and horizontal cell marker calbindin [19] in the P0 retina. S-opsin, RXRγ, and calbindin were colocalized with p27, indicating p27 expression in cones and horizontal cells (Fig. 1b). We further conducted double immunofluorescence for p27 and various cell type-specific markers at P21. p27 immunoreactivity was detected in Brn3+ ganglion cells [20], syntaxin + amacrine cells [21], Sox9+ Müller glia [22], Chx10+ bipolar cells [23], and RXRγ + cones while calbindin + horizontal cells lacked p27 immunolabeling (Fig. 1c). The p27 staining throughout the thickness of the outer nuclear layer (ONL) indicated p27 expression in rods (Fig. 1a and c). Altogether, p27 was detected in all postmitotic cell types in the mature mouse retina except horizontal cells, which expressed p27 only transiently during development.

### Ectopic division of bipolar cells and Müller glia in the p27KO retinas

**Fig. 2:**
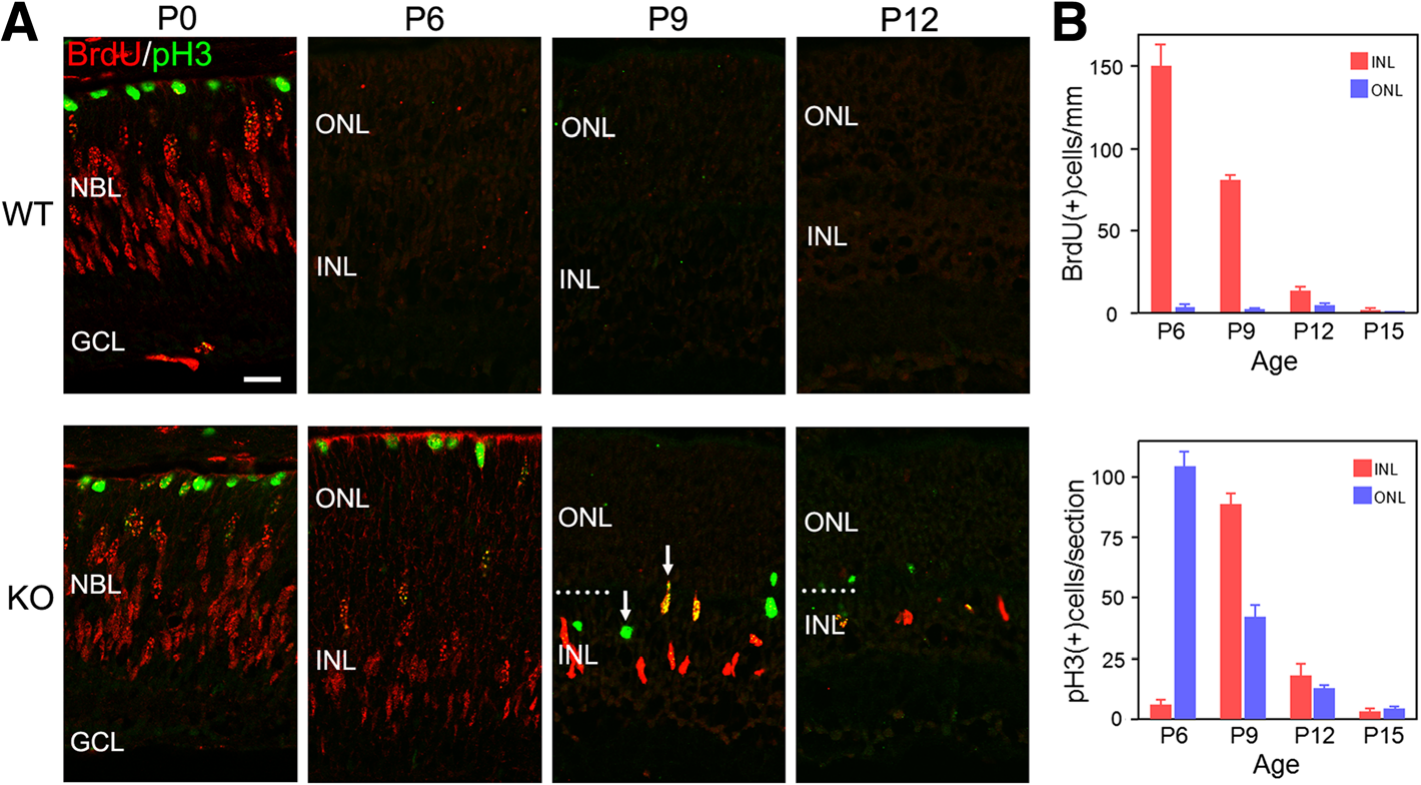
Ectopic cell division in the absence of p27. **a** Double immunofluorescence for BrdU and phospho-histone H3 (pH3) in the wild-type (WT) and p27 knockout (KO) retinas at different postnatal ages. The central retinal regions are shown. BrdU+ and pH3+ cells are observed only in the KO retinas at P6 and thereafter. Note pH3+ cells are ectopically positioned in the inner nuclear layer (INL) at P9 (arrows). GCL, ganglion cell layer; NBL, neuroblastic layer; ONL, outer nuclear layer. Dotted lines indicate the border between the ONL and INL. Scale bar = 20 μm. **b** Quantification of BrdU+ and pH3+ cells in the KO retinas. Each bar represents the mean ± SEM (*n* = 3 per age)

We next assessed the effects of p27 deletion on retinal cell proliferation during development. Wild-type (WT) and p27KO (KO) mice of various postnatal ages were labeled with BrdU for two hours prior to sacrifice, and retinal sections double-labeled for BrdU and phospho-histone H3 (pH3) to locate S and M phase cells, respectively. At P0, the patterns of BrdU and pH3 staining were similar between the WT and KO retinas (Fig. 2a). In both genotypes, BrdU+ S-phase cells were located in the inner neuroblastic layer, whereas pH3+ M-phase cells resided at the outer retinal margin. At P6 and later, division had virtually ceased in the central region of the WT retina while many BrdU+ and pH3+ cells were found throughout the KO retina (Fig. 2a and b). At P6, in the KO retinas, BrdU+ cells were located in the developing INL and pH3+ cells arranged at the outer margin of the retina, in a pattern similar to the previous stages (Fig. 2a and b). At P9, however, many pH3+ cells were found in the INL or the inner part of the ONL, away from the outer retinal margin where M phase cells normally occur (Fig. 2a and b). Proliferation was much less prominent at P12 and later stages (Fig. 2a and b) and no division was detected at P21 (data not shown).

**Fig. 3:**
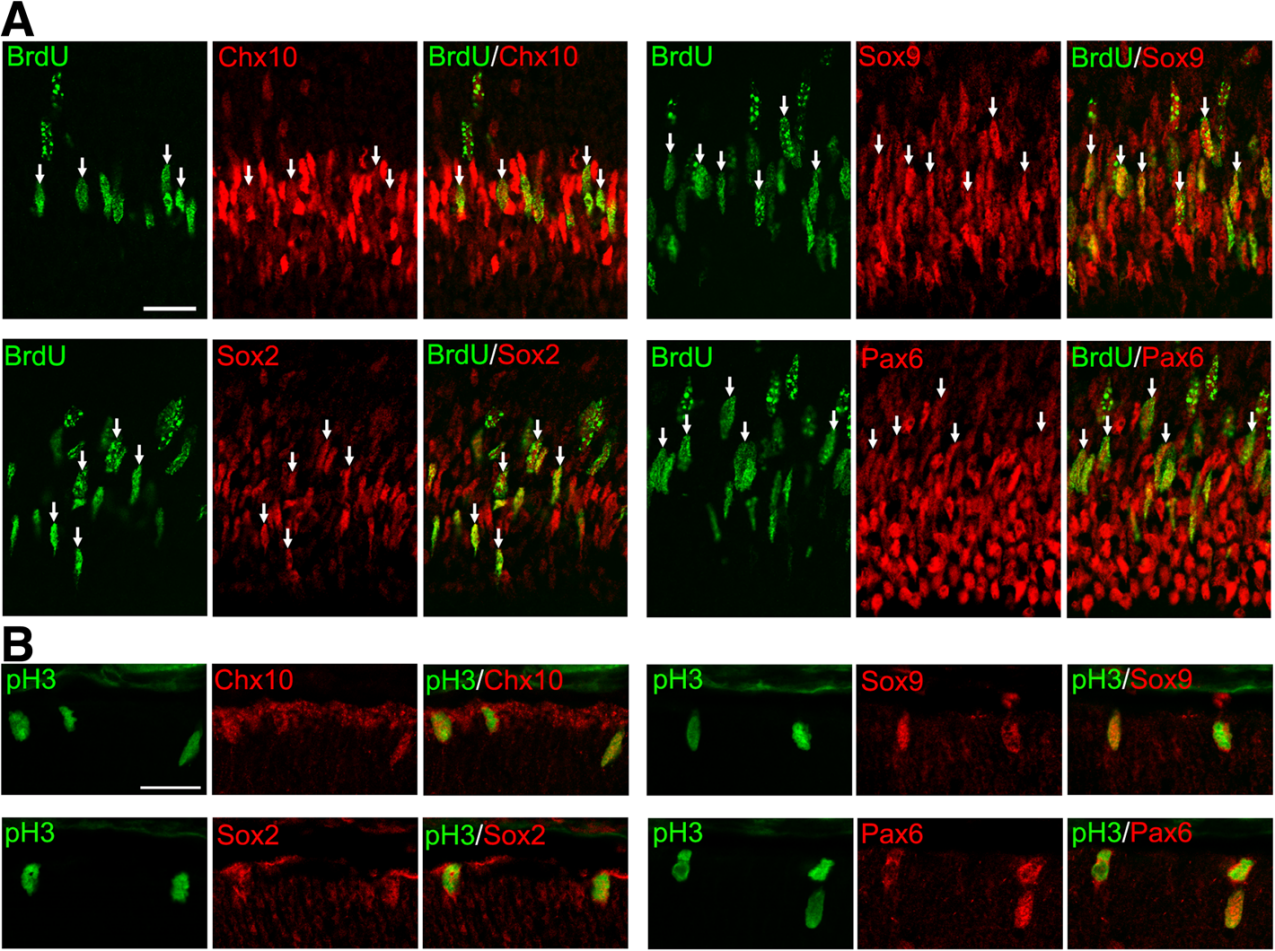
Progenitor characteristics of the proliferating cells in the p27 knockout retinas at P6. Double immunofluorescence for BrdU (**a**) or phospho-histone H3 (pH3) (**b**) with progenitor markers (Chx10, Sox9, Sox2, and Pax6) showing colocalization (arrows). Scale bars = 20 μm

**Fig. 4:**
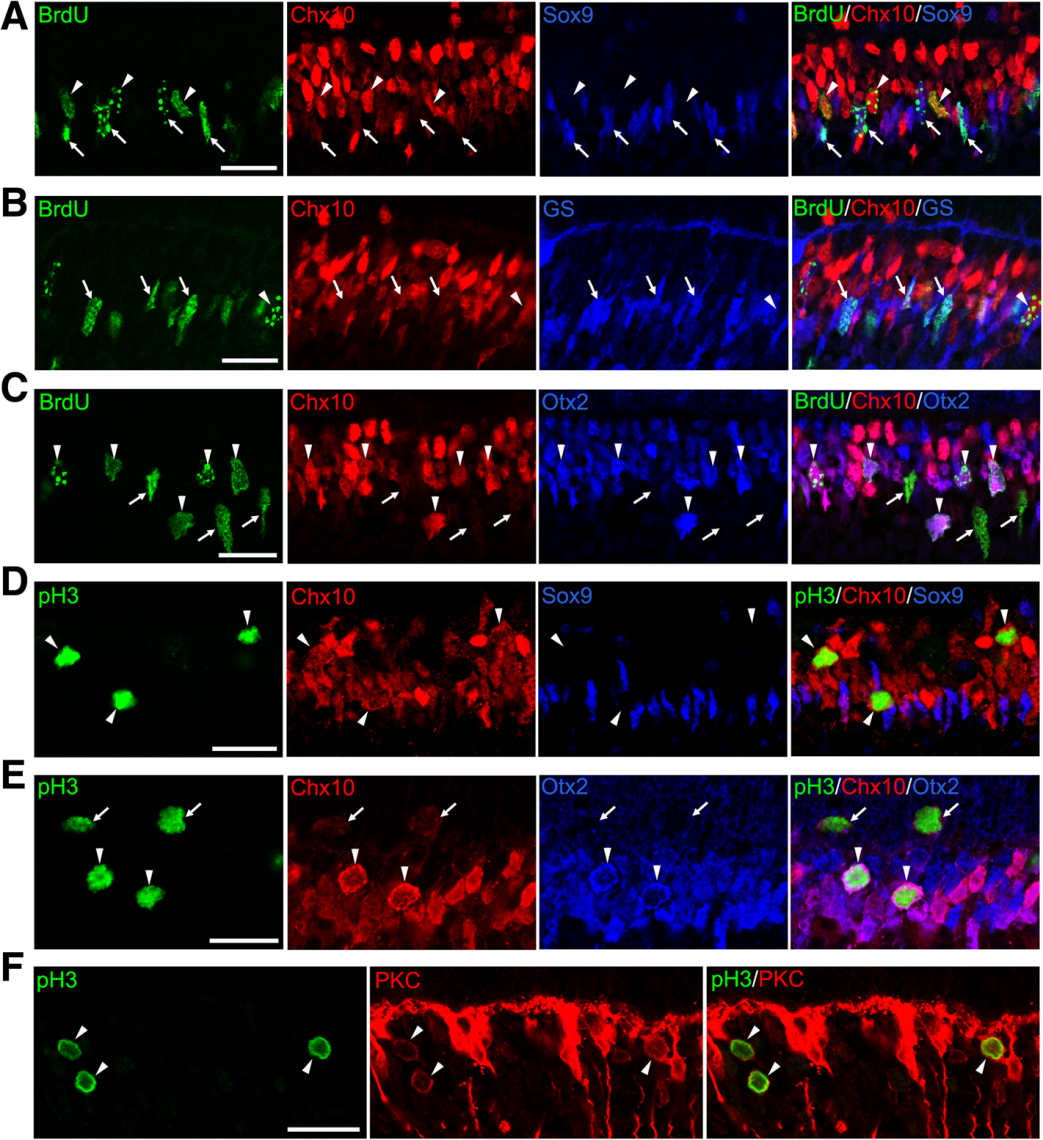
Ectopic cell cycle reentry of bipolar cells and Müller glia in the p27 knockout retinas at P9. **a** Triple immunofluorescence for BrdU, Chx10, and Sox9. Arrows indicate BrdU+ cells stained weakly for Chx10 and intensely for Sox9 (Müller glia) while arrowheads denote BrdU+ cells which are intensely Chx10+ and Sox9- (bipolar cells). **b** Triple immunofluorescence for BrdU, Chx10, and glutamine synthetase (GS). Arrows indicate BrdU+, weakly Chx10+ and GS+ cells (Müller glia). Arrowhead shows BrdU+, intensely Chx10+, and GS- cell (bipolar cell). **c** Triple immunofluorescence for BrdU, Chx10, and Otx2. Some BrdU+ cells are weakly Chx10+ and Otx2- (arrows, Müller glia) while others are intensely positive for both Chx10 and Otx2 (arrowheads, bipolar cells). **d** Triple immunofluorescence for phospho-histone H3 (pH3), Chx10, and Sox9. Arrowheads indicate ectopic M-phase cells strongly positive for Chx10, but negative for Sox9 (bipolar cells). **e** Triple immunofluorescence for pH3, Chx10, and Otx2. Arrows denote pH3+ cells stained weakly for Chx10 and negative for Otx2 (Müller glia). Arrowheads indicate pH3+/Chx10+/Otx2+ cells (bipolar cells). **f** Double immunofluorescence for pH3 and PKCα (PKC) showing colocalization (arrowheads, bipolar cells). Scale bar = 20 μm

The above results are consistent with the previous observations that the period of proliferation is extended in the KO retinas [8, 9]. However, we unexpectedly observed many ectopic M-phase cells in the inner retina at P9, but not at P6 or earlier, suggesting that proliferating cells at P9 are phenotypically different from those at P6. To more closely characterize cell proliferation in the KO retinas, we performed double or triple staining for proliferation markers (BrdU and pH3) in combination with retinal progenitor and differentiation markers. At P6, virtually all BrdU+ cells expressed the progenitor markers Chx10, Sox9, Sox2 and Pax6 [24] (Fig. 3a). PH3+ cells were also colabeled with these progenitor markers (Fig. 3b). These data are consistent with the progenitor phenotype of proliferating cell population in the KO retina at P6. However, all these progenitor markers (Chx10, Sox9, Sox2 and Pax6) are expressed not only by retinal progenitors but also by Müller glial cells [25]. To test the possibility that Müller glia had reentered the cell cycle in the absence of p27, expression of glutamine synthetase (GS), a Müller glia marker [25], was analyzed. No detectable signals were obtained in the KO retina at P6 (data not shown), further supporting the progenitor phenotype of dividing cells. Yet, we could not exclude the possibility that they were immature Müller glia without detectable expression of mature Müller markers. In contrast to the P6 retinas, at least three different proliferating populations were identified at P9, based on the expression of Chx10 and Sox9. Triple staining for BrdU, Chx10 and Sox9 revealed that about a half of BrdU+ cells were weakly labeled for Chx10 while intensely positive for Sox9 (46.6%, 308 cells per 661 BrdU+ cells counted, *n* = 3) suggesting that they were progenitor cells or proliferating Müller glia (Fig. 4a). These cells were shown to be GS+ (Fig. 4b), indicating their identity as Müller glia rather than progenitors. The other half of BrdU+ cells were intensely positive for Chx10 but were negative for Sox9 (48.9%, 323 cells per 661 BrdU+ cells counted, *n* = 3), consistent with the characteristics of bipolar cells (Fig. 4a). Only 4.5% of BrdU+ cells were Chx10−/Sox9- and thus classified as non-bipolar/non-Müller cells. We also conducted triple labeling for BrdU, Chx10, and Otx2, another bipolar marker [26]. Again, approximately half of BrdU+ cells (49.3%, 176 cells per 357 BrdU+ cells counted, *n* = 2) were colabeled with both Chx10 and Otx2, confirming their bipolar identity (Fig. 4c). Similarly, some pH3+ cells were labeled for both Chx10 and Sox9 (data not shown) while others were labeled for Chx10 and Otx2, but not for Sox9 (Fig. 4d and e). Of note, most M-phase cells ectopically positioned in the INL were the latter cell type displaying the bipolar phenotype. To further verify the bipolar identity, we carried out double staining for pH3 and a mature bipolar cell marker PKCα [27]. A significant proportion of ectopic pH3-positive cells (27.9%, 48 cells per 172 pH3+ cells counted, n = 2) were also labeled for PKCα. (Fig. 4f). Taken together, the expression patterns of key markers and the ectopic position of M-phase cells indicate that the proliferation in the KO retina at P9 is due to aberrant division of differentiating Müller glia and bipolar cells, but not a simple extension of progenitor proliferation.

### Fate of proliferating cells in the p27KO retinas

**Fig. 5:**
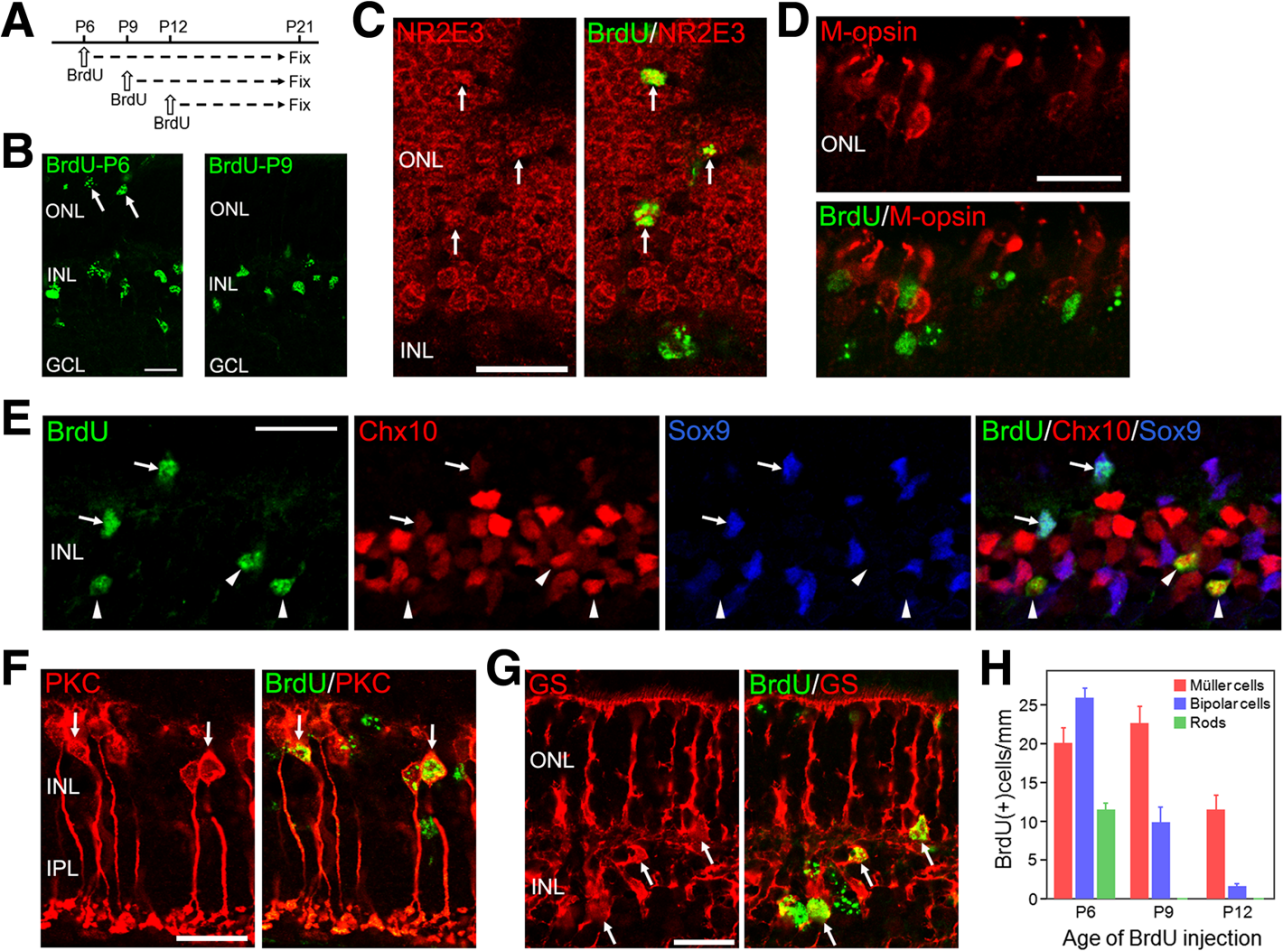
The fate of dividing cells in the p27 knockout retinas. **a** Experimental scheme of BrdU incorporation assays indicating the postnatal stages of BrdU injection and sacrifice. **b** Single staining for BrdU in the P21 retinas treated with BrdU at P6 and P9. Arrows indicate positive cells in the outer nuclear layer (ONL). **c** P21 retina (BrdU injected at P6) double-stained for BrdU and the rod marker NR2E3 showing colocalization (arrows). **d** P21 retina (BrdU injected at P6) double-stained for BrdU and the cone marker M-opsin. No colabeling is observed. **e** Triple immunofluorescence for BrdU, Chx10 and Sox9 in the P21 retinas injected with BrdU at P6. Arrows indicate BrdU+ Müller glia labeled weakly for Chx10 and intensely for Sox9. Arrowheads denote BrdU+ bipolar cells expressing Chx10 but not Sox9. **f** P21 retina (BrdU injected at P6) double-labeled for BrdU and the bipolar marker PKCα (PKC) showing colocalization (arrows). **g** P21 retina (BrdU injected at P6) double-labeled for BrdU and the Müller marker glutamine synthetase (GS) showing colocalization (arrows). **h** Quantification of the cell types which were in the last S-phase at the time of BrdU injection. Rods were identified by NR2E3 labeling, and bipolar and Müller cells were determined based on the expression of Chx10 and Sox9. Each bar represents the mean ± SEM (*n* = 3 per stage). GCL, ganglion cell layer; INL, inner nuclear layer. Scale bars = 20 μm

We next sought to determine the fate of dividing cells in the KO retinas by BrdU incorporation assays. KO mice were injected with BrdU at P6, P9 or P12 and allowed to survive until P21 (Fig. 5a). Cells which were in their last S-phase at the time of BrdU injection were expected to show intense BrdU labeling, and their identity was analyzed by double or triple staining for BrdU and cell-specific markers. Strongly BrdU-labeled cells were observed both in the INL and ONL when BrdU was injected at P6 (Fig. 5b). BrdU+ cells in the ONL were identified as rods based on the expression of the rod marker NR2E3 [28] (Fig. 5c and h) and the absence of the cone markers S- and M-opsins (Fig. 5d and data not shown). BrdU+ cells in the INL or the inner portion of the ONL were identified as Müller glia or bipolar cells by expression patterns of Chx10, Sox9, PKCα, and GS (Fig. 5e, f, g, and h). On the other hand, cells which incorporated BrdU at P9 or P12 were found mostly in the INL (Fig. 5b) and identified as bipolar cells and Müller glia (Fig. 5h).

### Aberrant cell cycle reentry of cones in the p27KO retinas

**Fig. 6:**
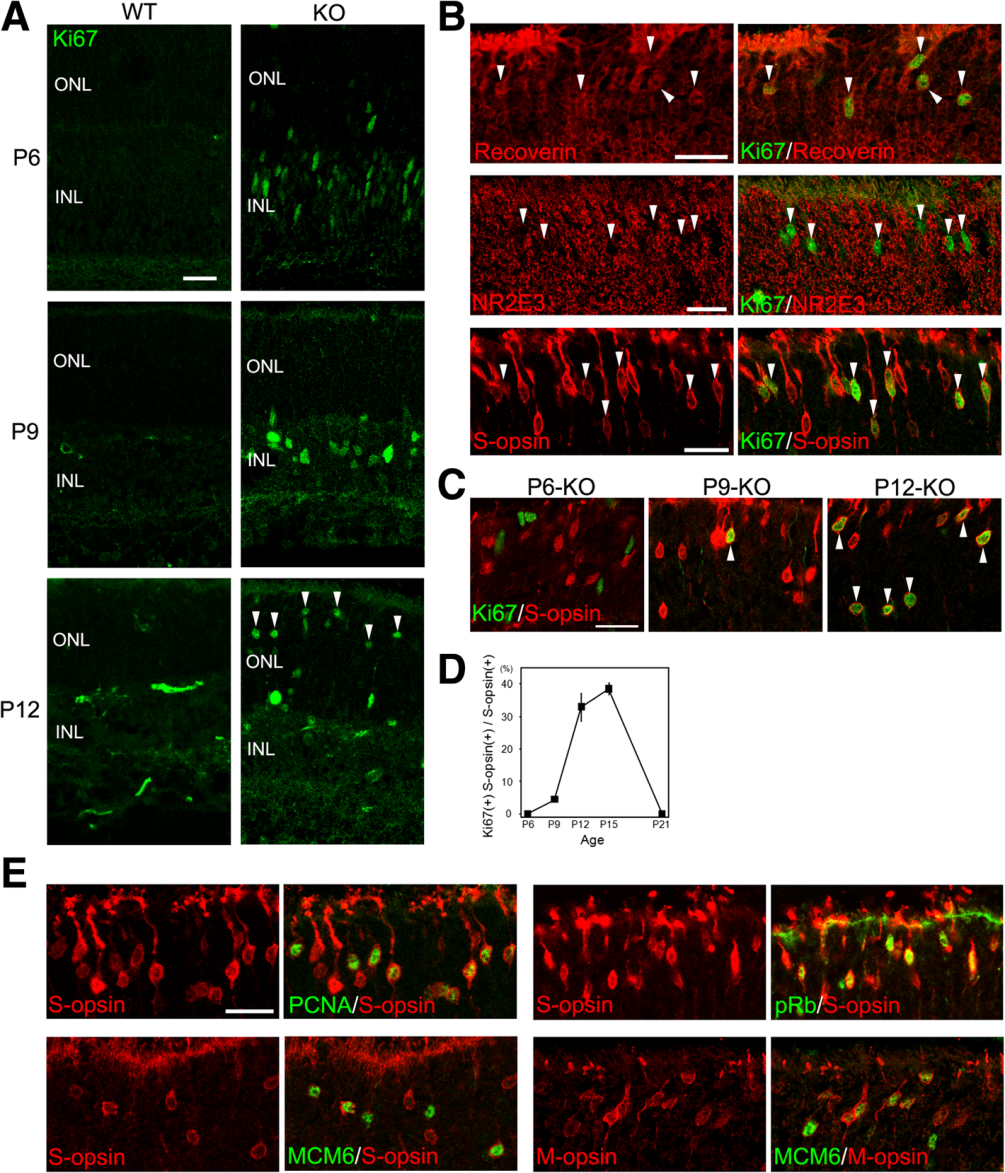
Aberrant cell cycle reentry of cones in the p27 knockout (KO) retinas. **a** Immunofluorescence for Ki67 in the WT and KO retinas at P6 and later stages showing proliferating cells only in the KO retinas. Arrowheads indicate Ki67+ cells in the outer nuclear layer (ONL) of the KO retina at P12. INL, inner nuclear layer. **b** The ONL of the KO retina at P12 immunolabeled for Ki67 in combination with the photoreceptor marker recoverin, rod marker NR2E3, and cone marker S-opsin. Note colocalization of Ki67 with recoverin and S-opsin, but not with NR2E3 (arrowheads). **c** Double immunofluorescence for Ki67 and S-opsin in the KO retinas at P6 and later stages. Ki67+/S-opsin + cones are shown by arrowheads. **d** Quantification of Ki67+ cones in the KO retinas. Graph data represent the means ± SEM (n = 3 per stage). **e** Cones identified by S-opsin or M-opsin immunoreactivity express proliferation markers PCNA, phospho-Rb, and MCM6 in the KO retinas at P12. Scale bars = 20 μm

The presence of ectopic division in the KO retinas prompted us to examine Ki67 expression, which labels all phases of the cell cycle. While no Ki67+ cells were observed in the central portion of the WT retinas at P6 and later stages, many positive cells were found in the KO retinas (Fig. 6a), consistent with the results obtained using BrdU and pH 3 as cell cycle markers. At P6 and P9, most Ki67+ cells occurred in the INL, similar to the pattern of BrdU staining. Unexpectedly, however, many Ki67+ cells were observed in the ONL of the KO retinas at P12 (Fig. 6a). To test the possibility that the Ki67+ cells in the ONL were photoreceptors, we carried out double staining for Ki67 and several photoreceptor markers. Ki67 was colocalized with the photoreceptor marker recoverin and cone marker S-opsin, but not with the rod marker NR2E3 (Fig. 6b), indicating their cone identity. We next quantified the proportion of cones expressing Ki67 in the KO retinas at different ages. At P6, there were many S-opsin + cones in the ventral half of the retina, but none of them were labeled for Ki67 (Fig. 6c and d). However, approximately 5% of S-opsin + cones expressed Ki67 at P9, and 30–40% of S-opsin + cones were labeled for Ki67 at P12 and P15 (Fig. 6c and d). No S-opsin + cones expressed Ki67 at P21 (Fig. 6d). We also analyzed other cell cycle markers including PCNA, phospho-Rb (pRb), MCM6, BrdU and pH3 for colocalization with cone opsins. A significant fraction of S-opsin + or M-opsin + cones expressed PCNA, pRb and MCM6 (Fig. 6e) while few cones were labeled for BrdU and none expressed pH3 (data not shown).

**Fig. 7:**
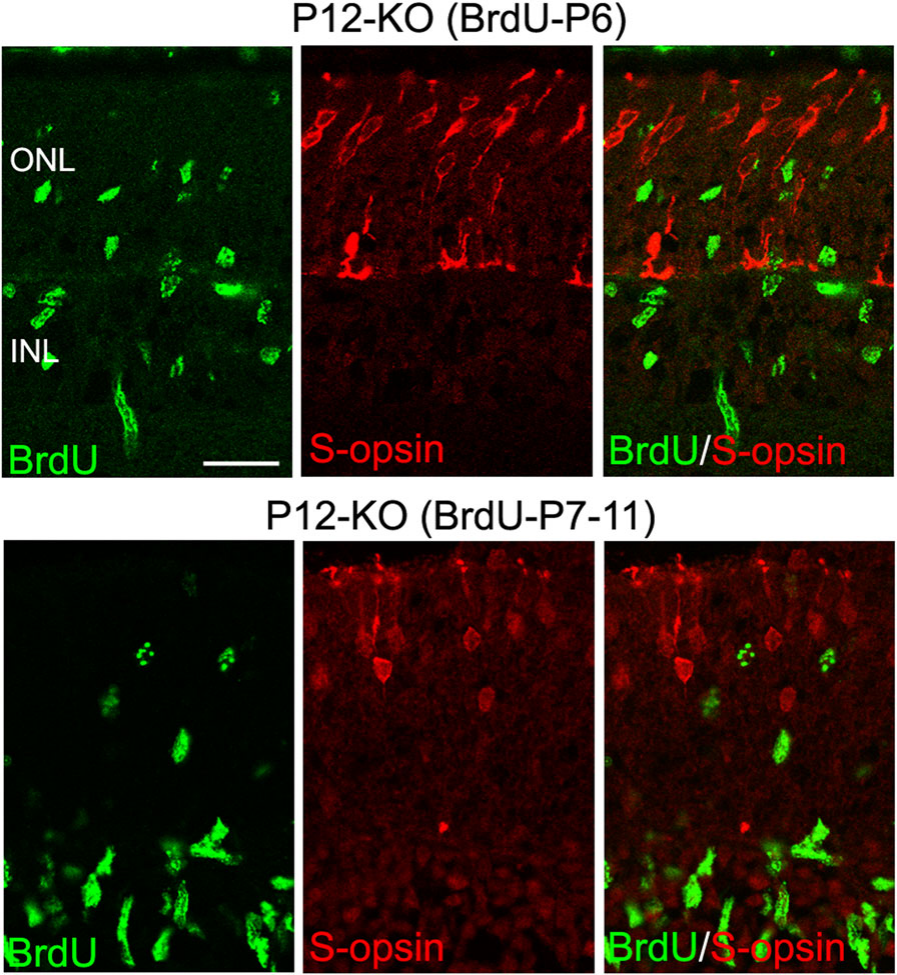
Cones are not generated de novo during the period of extra proliferation in the p27 knockout (KO) retinas. KO mice were treated with BrdU at P6 or daily from P7 to P11, and the fate of proliferating cells analyzed at P12. Double labeling for BrdU and S-opsin shows no colocalization. ONL, outer nuclear layer; INL, inner nuclear layer. Scale bar = 20 μm

Because cones are normally generated during the embryonic period [29], these results suggest that p27-dificient cones underwent aberrant cell cycle reentry after a long quiescent period. However, it was also possible that proliferating cones were generated de novo during the period of extra proliferation. To test this possibility, we labeled the proliferating cell population in the KO retinas at P6 with BrdU and examined their fate at P12, but none of the BrdU+ cells expressed S-opsin (Fig. 7). We also traced the fate of proliferating cells by daily injection of BrdU from P7 to P11 to find no evidence of de novo generation of cones (Fig. 7). Thus, cones seemed to differentiate normally in the absence of p27 until they suddenly reenter the cell cycle after a quiescence of several weeks.

### Differentiation and survival of cones are impaired in the p27KO retinas

**Fig. 8:**
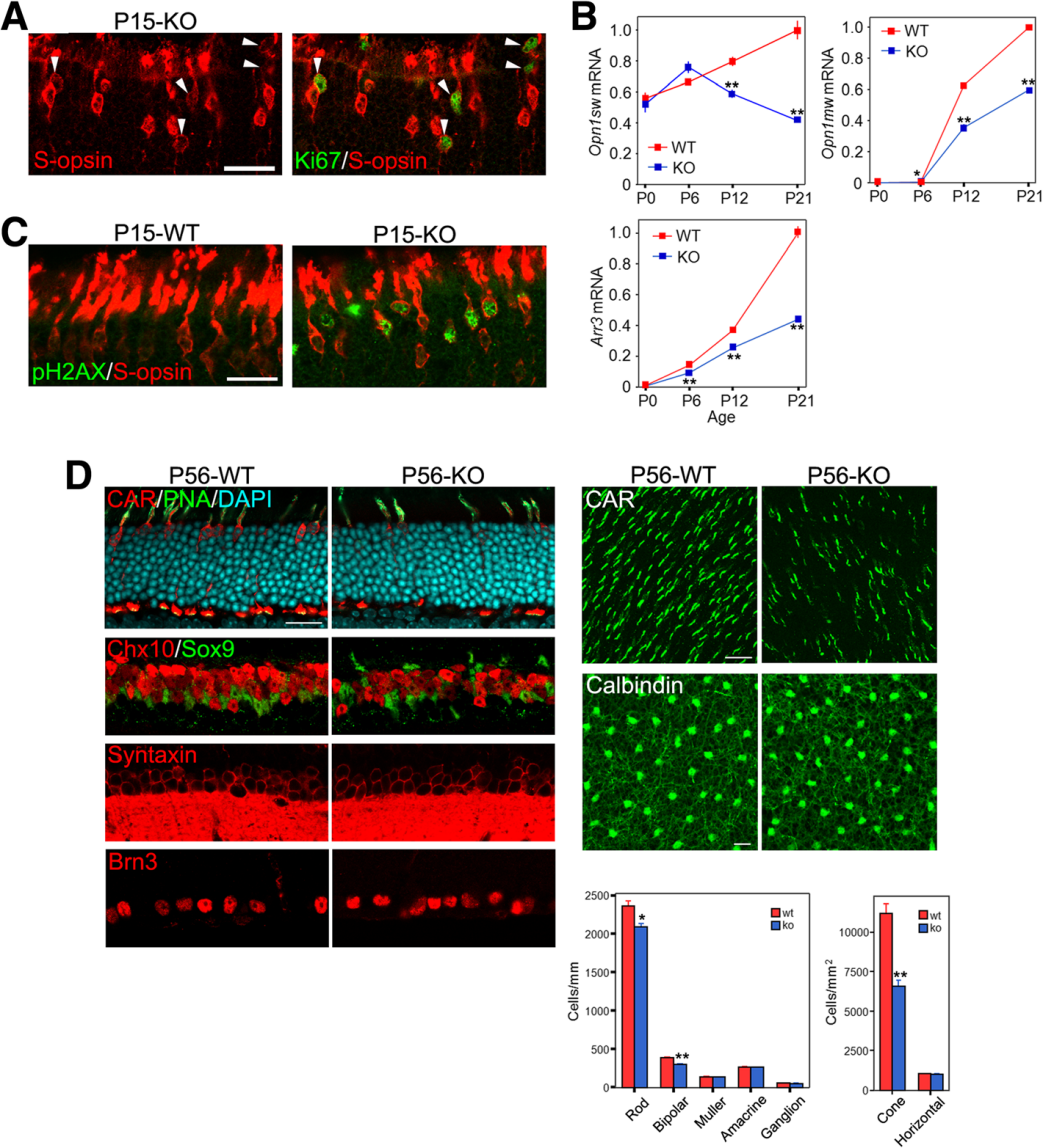
Impaired differentiation and survival of cones in the p27 knockout (KO) retinas. **a** Double immunofluorescence for Ki67 and S-opsin in the KO retina at P15. Note relatively weak S-opsin labeling in the Ki67+ cones (arrowheads). **b** Quantitative RT-PCR analyses of cone gene expression in the WT and KO retinas during postnatal development. The transcript levels are expressed relative to WT at P21 after normalized to *Gapdh* levels. Each value represents the mean ± SEM (*n* = 3 per stage and genotype). *P < 0.05, **P < 0.01, Student’s t test. **c** Double immunofluorescence for phospho-H2AX (pH2AX) and S-opsin in the WT and KO retinas at P15. Note pH2AX+ cones in the KO retina. **d** Quantification of retinal cell types in the WT and p27KO retinas at P56. Retinal sections or whole mounts were immunolabeled for cell-specific markers. Note a significant reduction in cone number in the KO retina. The number of rods and bipolar cells are also mildly reduced. Bars represent the mean ± SEM (*n*= 3 per genotype). *p < 0.05, **p < 0.01, Student’s t test. Scale bars are 20 μm

We closely examined S-opsin+ cones in the KO retinas at P15, when the highest proportion of cones expressed cell cycle markers, such as Ki67. We found that S-opsin immunoreactivity in Ki67+ cones was weaker compared to Ki67- cones (Fig. 8a). We next examined expression of cone-specific genes in the WT and KO retinas during postnatal development by quantitative RT-PCR. There were no significant differences in the levels of *Opn1sw* (S-opsin) mRNA between genotypes at P0 and P6, while its expression was significantly decreased in the KO retinas compared to WT at P12 and P21 (Fig. 8b). Likewise, expression of *Opn1mw* (M-opsin) and *Arr3* (cone arrestin) in the KO retinas was significantly lower compared to WT at P6 and later stages (Fig. 8b). These results lead to the hypothesis that differentiation and/or survival of cones are impaired in the KO retinas, possibly due to their aberrant cell cycle reentry. To test this, we examined cell death by TUNEL assays. Although significantly more apoptotic cells were observed in the ONL of the KO retinas compared to WT at P12 and P21 (data not shown), the attempt to determine the identity of apoptotic cells by combining TUNEL assays with immunolabeling was not successful, possibly due to degradation of cell type markers in end-stage apoptotic cells detected by TUNEL assays. We then conducted double staining for S-opsin and phospho-histone H2AX (pH2AX), an early marker of DNA damage [30, 31]. Notably, a significant proportion of S-opsin+ cones were labeled for pH2AX in the KO retinas at P15, but not in the WT retinas (Fig. 8c).

It has been reported that p27 loss does not affect the proportion of retinal cell types [8, 9]; however, these studies did not examine the number of cones. Thus, we quantified the number of retinal cell types, including cones, by immunohistochemistry for cell-type specific markers in the retinas of 8-week-old WT and KO mice (Fig. 8d). Because of their sparsity, the numbers of cones and horizontal cells were assessed by whole mount staining while other cell types were evaluated on sections. Cones, horizontal cells, amacrine cells and ganglion cells were identified by immunoreactivity for cone arrestin, calbindin, syntaxin, and Brn3, respectively. Double staining for Chx10 and Sox9 were employed to identify bipolar cells (Chx10+/Sox9-) and Müller cells (Chx10+/Sox9+). Rods were counted as the number of DAPI-labeled nuclei in the ONL subtracted by the number of cones stained for cone arrestin and PNA. Intriguingly, the number of cones in the KO retinas was decreased by approximately 40% compared to the WT retina. The numbers of rods and bipolar cells were also mildly, but significantly, reduced in the KO retinas compared to WT. There were no significant differences in the number of other cell types between genotypes.

## Discussion

Previously, two groups reported that p27 loss induces prolonged proliferation of retinal progenitors, suggesting that p27 controls the timing of cell cycle exit of retinal progenitors [8, 9]. Indeed, we observed persistent proliferation in the p27-deficient retinas at P6 when proliferation in the central retina has normally ceased. Proliferating cells observed at P6 have molecular characteristics of retinal progenitors and were able to differentiate into multiple cell types including rods, bipolar cells and Müller glia. Thus, in agreement with the previous reports, our data showed that p27 loss perturbs the normal timing of progenitor cell cycle exit. However, in contrast with the previous reports, our data indicate that extra proliferation observed at P9 and later stages was not due to persistent progenitors, but due to aberrant division of differentiating cells. These dividing cells expressed differentiation markers of bipolar cells, Müller glia and cones together with proliferation markers. The discrepancy between our data and the previous findings may simply be explained by methodological differences. One study [9] analyzed cell proliferation only by single BrdU immunolabeling, which, without co-staining with differentiation markers, would not discriminate between progenitors and ectopic division of differentiating cells. The other study [8] used several proliferation markers including BrdU, PCNA and pH3 to assess proliferation, but proliferation was analyzed only at P11 or adult stages and double labeling with differentiation markers was not conducted. We employed a range of proliferation markers including BrdU, pH3, Ki67, PCNA, pRb and MCM6 to assess proliferation and performed careful marker analysis by double or triple immunolabeling for proliferation markers, progenitor markers and differentiation markers at various developmental time points, enabling us to discriminate ectopic cell cycle activity from progenitor proliferation.

That p27 loss induced aberrant cell cycle activity in differentiating cells suggests that p27 may be required to maintain the quiescence of postmitotic cells. This notion is consistent with the previous report that acute inactivation of p27 is sufficient to induce proliferation of adult Müller glia [32]. It was also recently shown that p27 loss promotes proliferation of Müller glia and retinal pigment epithelial (RPE) cells in the adult retina after photoreceptor damage [33]. Such a role for p27 has been described for other cell lineages such as pituitary cells [34], supporting cells in the cochlea [35] or cardiomyocytes [36], which undergo unscheduled cell cycle reentry in the absence of p27. Although previous reports have suggested that p27 is expressed only transiently in early postmitotic cells except Müller glia, which maintain p27 expression in the mature retina [8, 9], our data have shown persistent expression of p27 in most postmitotic retinal cells throughout development and in the adult, consistent with the role of p27 in the maintenance of postmitotic cells.

p27 may play a pivotal role in maintaining quiescence in bipolar cells, Müller glia and cones while it is dispensable for preventing cell cycle reentry in other cell types. The cell type-specific effects of p27 loss observed herein is unexpected given that virtually all postmitotic retinal cells express p27. Because p27 is known to act redundantly with other CDK inhibitors or Rb-like proteins [37–39], p27 loss may be compensated for by other cell cycle inhibitors in a cell type-specific manner. Interestingly, deletion of both p27 and p19^Ink4d^, but not single deletion of either CDK inhibitor, has been shown to induce ectopic cell cycle reentry of ganglion cells, amacrine cells and horizontal cells, suggesting that p27 and p19 may act redundantly to prevent ectopic division of these cell types [6]. These authors failed to find ectopic division of bipolar cells, Müller glia and cones, most likely because they characterized ectopic division only at P18, when ectopic proliferation of these cell types is no more detected in the p27-deficient retinas.

It has been reported that p27 loss does not affect the proportion of the major retinal cell types [8, 9]. However, these studies did not assess the number of cones in the p27-deficient retinas. We quantified the number of cones, as well as other major retinal cell types, and found that the number of cones in the p27-deficient retinas was decreased by approximately 40% compared to WT. The number of rods and bipolar cells were also modestly decreased while other cell types displayed no significant changes. The expression of S-opsin in the p27 mutants were normal at birth and P6, indicating that p27 loss does not affect the production and early differentiation of cones. However, subsequent reduction in cone number and the expression of cone-specific genes indicates that the p27-deficient retinas have a major defect in cone survival. Interestingly, both cell cycle reentry and DNA damage response (H2AX phosphorylation) in cones peaked at P15, suggesting a mechanistic association between these events. Of note, cell cycle reentry of cones increased drastically from P9 to P12 while H2AX phosphorylation was not detected until P15, indicating that cell cycle reentry precedes DNA damage responses in cones. Furthermore, S-opsin levels in cones expressing proliferation markers were lower compared to quiescent cones, suggesting that cones in the aberrant cell cycle were in the process of degeneration. All these data suggest that the aberrant cell cycle activity of cones is causally related to cone death.

Cones reentering the cell cycle expressed a variety of proliferation markers such as Ki67, PCNA, pRb and MCM6, but only few incorporated BrdU and none expressed pH 3. Thus, despite the activation of cell cycle machinery, most cones may fail to progress through the cell cycle and are prone to arrest or die. This contrasted markedly with ectopic proliferation of bipolar and Müller cells, many of which seemed to progress through S and M phases of the cell cycle as assessed by BrdU incorporation and pH 3 labeling. The mechanisms that determine the fate of these cell types remain unknown. Previous studies reported that inactivation of Rb/p107 in the retina induced ectopic division of all cell types, followed by death of ganglion cells, bipolar cells, rods and cones while other cell types were death-resistant [11, 12]. Thus, whether ectopic division is followed by cell death may depend on cell-specific intrinsic properties. Our data may indicate that bipolar cells and Müller glia have a greater tolerance for unscheduled DNA synthesis than cones. However, the modest reduction in bipolar cell number suggests that this cell type is not fully death-resistant in the context of p27 loss. Alternatively, differentiated cells may be more susceptible to death as a result of the conflict between differentiation and division compared to less differentiated immature cells. Bipolar cells and Müller glia are born during the first postnatal week [17], and ectopic division occurs only several days later. On the other hand, the generation of cones peaks at E13-E14 [29] and they reenter the cell cycle approximately three weeks after generated. Thus, the late onset of cell cycle events may contribute, at least in part, to the death-prone phenotype of p27-deficient cones. Finally, we cannot exclude the possibility that non-cell-autonomous mechanisms may be involved in the robust cell death of cones. The p27-deficient retina exhibits dysplasia of the ONL, characterized by disruption of the outer limiting membrane and displaced photoreceptor cells outside the outer limiting membrane [9, 40]. Another report has also suggested that the normal contact between photoreceptor outer segments and the RPE is substantially disrupted in the p27-deficient retina [41]. Thus, the disorganization of the ONL might affect the differentiation and/or survival of photoreceptors. Indeed, despite the absence of ectopic division of rods, rod number was modestly, but significantly, decreased in the mature mutant retina. Thus, disruption of normal microenvironment due to retinal disorganization may have triggered photoreceptor cell death.

## Conclusions

Although p27 is expressed in all differentiating cell types in the mouse retina, this CDK inhibitor is required to maintain the quiescence of specific cell types including bipolar cells, Müller glia, and cone photoreceptors while it is dispensable for preventing cell cycle reentry in other cells types. Moreover, p27 is required for normal differentiation and survival of cones. Our data provide new insights into the role of CDK inhibitors in the maintenance, survival, and degeneration of retinal cells.

## Additional file

Additional file 1: Table S1. Antibodies. **Table S2.** Primers. (DOCX 17 kb)

## Abbreviations

Calb: calbindin
CAR: cone arrestin
CDK: cyclin-dependent kinase
GCL: ganglion cell layer
GS: glutamine synthetase
INL: inner nuclear layer
KO: knockout
NBL: neuroblastic layer
ONL: outer nuclear layer
pH 3: phospho-histone H3
pH2AX: phospho-H2AX
PNA: peanut agglutinin
RT-PCR: reverse transcription-polymerase chain reaction
Syn: syntaxin
WT: wild type
