# Supplementary material for: Cell type-specific effects of p27KIP1 loss on retinal development
Source: Neural Dev. 2017 Sep 20;12:17. doi: 10.1186/s13064-017-0094-1 (PMC5607500; doi:10.1186/s13064-017-0094-1)
Supplement: Additional file 1: Table S1. — Antibodies. Table S2. Primers. (DOCX 17 kb) [file 13064_2017_94_MOESM1_ESM.docx]

**Table S1.** Antibodies

| Antibody | Dilution | Species | Source |
| --- | --- | --- | --- |
| BrdU | 1:500 | Mouse | Lab Vision MS1058B0 |
| Phospho-histone H3 | 1:1000 | Rabbit | Millipore 06-570 |
| Chx10 | 1:2000 | Sheep | Exalpha X1180P |
| Sox9 | 1:2000 | Rabbit | Millipore AB5535 |
| Pax6 | 1:2000 | Rabbit | Millipore AB5409 |
| Sox2 | 1:4000 | Rabbit | Millipore AB5603 |
| Glutamine synthetase | 1:5000 | Rabbit | Sigma G2781 |
| PKCα | 1:2000 | Mouse | Sigma P5704 |
| NR2E3 | 1:2000 | Rabbit | Shiming Chen, Washington Univ, St. Louis |
| Ki67 | 1:20 | Mouse | Dako M7248 |
| Otx2 | 1:5000 | Rabbit | Millipore AB9566 |
| Recoverin | 1:1000 | Rabbit | Millipore AB5585 |
| S-opsin | 1:500 | Goat | Santa Cruz SC14363 |
| M-opsin | 1:2000 | Rabbit | Millipore AB5405 |
| RXRγ | 1:400 | Rabbit | Santa Cruz SC555v |
| Phospho-histone H2AX | 1:100 | Rabbit | Cell Signaling 9718 |
| Cone arrestin | 1:500 | Rabbit | Cheryl M. Craft, Doheny Eye Institute, Los Angeles |
| Syntaxin | 1:200 | Mouse | Sigma S0664 |
| Brn3 | 1:50 | Goat | Santa Cruz SC6026 |
| Calbindin | 1:2000 | Rabbit | Millipore AB1778 |
| p27 | 1:1000 | Mouse | BD 610241 |
| Phospho-Rb | 1:1000 | Rabbit | Cell Signaling 9308 |
| PCNA | 1:200 | Mouse | Dako M0879 |
| MCM6 | 1:10000 | Goat | Santa Cruz SC9843 |

**Table S2.** Primers

| Gene | Forward | Reverse |
| --- | --- | --- |
| *Gapdh* | 5’-TGAAGGTCGGTGTCAACGGATTTGGC-3’ | 5’-CATGTAGGCCATGAGGTCCACCAC-3’ |
| *Opn1sw* | 5’-TCACGGATACTTCCTCTTTGGT-3’ | 5’-CAGATGACAACGTAGCGTTCA-3’ |
| *Opn1mw* | 5’-TTGCTGACCTAGCAGAGACCA-3’ | 5’-AGCCTTCAATGACACACAGAG-3’ |
| *Arr3* | 5’-AAGAAGACTAGCTCCAATGGGA-3’ | 5’-AACAAGGACGACTCCATCAATG-3’ |
